# The Importance of Studying Factors That Affect the In Vitro Evaluation of Semen Quality to Predict Potential Fertility in Males

**DOI:** 10.3390/biology12020235

**Published:** 2023-02-02

**Authors:** Miguel Angel Silvestre, Carles Soler, Eva Mocé, Eduardo R. S. Roldan, Jesús L. Yániz

**Affiliations:** 1 Departamento de Biología Celular, Biología Funcional y Antropología Física, Universitat de València, 46100 Burjassot, Spain; 2 Centro de Investigación y Tecnología Animal, Instituto Valenciano de Investigaciones Agrarias, 12400 Segorbe, Spain; 3 Department of Biodiversity and Evolutionary Biology, Museo Nacional de Ciencias Naturales (CSIC), 28006 Madrid, Spain; 4 BIOFITER Research Group, Institute of Environmental Sciences (IUCA), University of Zaragoza, 22071 Huesca, Spain

The presence of sub-fertile or infertile males in farms or artificial insemination (AI) centres has a great impact on the reproductive and economic performance of the livestock industry. Assessment of the field fertility of males or ejaculates (or seminal samples from experimental protocols) with a large number of AI procedures can take a long time and involves high costs. The early detection of these males or semen samples with low-potential fertility by means of in vitro analysis is extremely valuable. While infertile male detection may be more or less evident, the detection of sub-fertile males requires procedures of in vitro evaluation of sperm quality to be as optimized. Moreover, to evaluate the effect of cooled storage or cryopreservation, it is essential to have adequate procedures for in vitro evaluation, to know the real impact of these techniques and to improve them. Most published papers reporting optimizing procedures for in vitro assessment of sperm focus on human, laboratory animal or livestock species with important economic weight. However, the description of semen quality parameters in other wild or less studied, but no less important, species (e.g., bees) is vital for animal biodiversity conservation programs.

Despite improvements in recent years, the predictive ability of the in vitro analysis of semen continues to be limited. Optimization and standardization of procedures for sperm quality parameter evaluation is essential in order to obtain reliable data. Among sperm parameters, total or progressive motility and kinetics are some of the most frequently utilized and those with a closer correlation with fertility. Other important parameters include sperm viability, morphology, DNA fragmentation, and acrosome integrity. In this context, the use of automated technologies is advisable as they are more objective and are capable for analysing a huge number of cells per sample in a short time. Examples are computer-assisted sperm analysis (CASA) systems for motility, morphometry and flow cytometry for other sperm parameters using several fluorescent probes. However, applied procedures are not completely optimized or standardized. In addition, in vitro methodologies to assess the in vitro quality of sperm which have been optimized for one animal species can require adaptation or modification to other species due to differing sperm morphologies and physiology between species.

This Special Issue of *Biology* entitled “Factors Affecting in Vitro Assessment of Sperm Quality” focuses on the most frequently used and advanced procedures for in vitro sperm evaluation, mainly sperm motility, and their application in studies on the conservation of semen doses. The Special Issue contains six papers, including one review paper. There are two papers for each of the three main topics.

Topic 1: optimization of sperm motility evaluation with a new Open CASA system and the statistical analysis of CASA technology parameters.

CASA technology comprises both commercial and open systems. Commercial systems started to appear about 40 years ago and several brands can guarantee the quality of their results; subsequently, they are used in many laboratories around the world. More recently, Open CASA software has increased the accessibility of the technology, allowing scientists to improve the analysis capabilities of the software. In fact, similar to other open software, Open CASA is easier to improve through the collaboration of different scientific groups. In this Special Issue, three new modules for the analysis of chemotactic sperm accumulation, sperm functionality (based on fluorescent stained cells), and sperm concentration are presented. In particular, the study of chemotaxis provides a very useful tool for the research into how sperm reacts to a variety of substances, making such knowledge available for future applications in the field of fertility. Of similar importance is a novel module that can automatically measure one technique for the evaluation of different sperm functional parameters. As a result of the introduction of these three new modules, Open CASA (hosted at Github) is now the most powerful CASA system [1].

Independent of the CASA technology used, the data obtained (kinematic, morphometric, etc.) has for a long time been evaluated with classical statistics simply using the comparison of results following ANOVA (or non-parametric analysis) to differentiate among species, brands, or treatments. This approach continues to be used by some laboratories, but it may be considered much too limited. During the last 20 years, a new approach has been introduced with the analysis of sperm subpopulations (SPs). It has been shown that the former model, that considered the ejaculate as one population of spermatozoa competing in some way to arrive at the oocyte, is not satisfactory. Rather than considering the population as a whole, sperm cells in each ejaculate have been shown to have clearly defined differences. However, the mathematical calculation of sperm SPs is not enough to indicate their biological significance. New approaches such as Bayesian statistics can help to determine how SPs influence the fertility of samples [2].

Topic 2: importance of in vitro sperm parameters to assess the effects of semen conservation.

In the first work relating to this second topic, Peris-Frau et al. [3] analysed the effects of cryopreservation and an artificial capacitation treatment on ram sperm, studying the in vitro sperm parameters, such as motility and kinetics, mitochondrial membrane potential and tyrosine phosphorylation, measured by the CASA system and flow cytometry. They showed how capacitation produced a significant change in the distribution of ram sperm subpopulations (after cluster analysis) depending on whether the samples were frozen-thawed or fresh. In particular, two sperm subpopulations, SP1 and SP4, corresponding to inactive and low MMP spermatozoa and sperm with fast non-linear motility, respectively, were the most affected after capacitation, depending on whether they were fresh or frozen samples.

In the second work, Sadeghi et al. [4] examined the effect of goat sperm concentration, refrigeration temperature and time storage, studying in vitro sperm parameters such as motility and kinetics, response to oxidative stress, mitochondrial membrane potential measured by the CASA system and flow cytometry, and sperm DNA fragmentation. Three experiments were carried out to evaluate the effects of two temperatures (5 and 17 °C) and four sperm concentrations for 48 h of semen storage. They found semen refrigeration at 5 °C maintained higher values of motility than at 17 °C in a milk-based extender. Moreover, the diminution of sperm concentration in cooled storage did not seem to improve the semen quality of the samples.

Topic 3: advances in the characterization and quality analysis of sperm in aquatic and aerial species: from dolphins to bees.

In the first work under this heading, Fuentes-Albero et al. [5] present a novel complete description of sperm morphometry and morphometric subpopulations in the bottlenose dolphin (*Tursiops truncatus*) and the influence of blood testosterone and refrigeration on the sperm morphometry. Two different sperm subpopulations, which differed between males, were described. The levels of blood testosterone and the storage period in refrigeration had a substantial impact on some sperm morphometric traits (e.g., midpiece and flagellum length), while they were barely affected by the refrigeration temperature.

In the second work, Yániz et al. [6] provide a comprehensive review of sperm quality in vitro analysis in honey bee (*Apis mellifera*) drones. The main seminal parameters, such as sperm concentration, motility, viability and mitochondrial membrane potential are reviewed. This review highlights the importance of improving current sperm quality in vitro analysis and proposes future research lines. Given the particular morphology and physiology of honey bee sperm, there is a need for the development of technologies specifically adapted to bees. Despite advances in recent years, the development of more sophisticated and objective methods to evaluate sperm quality still remain to be developed, mainly in the case of the honey bee.

Finally, we would like to acknowledge all the authors who have participated in this Special Issue by contributing their works, and we would also like to thank the anonymous reviewers.
